# Systemic interindividual DNA methylation variants in cattle share major hallmarks with those in humans

**DOI:** 10.1186/s13059-024-03307-6

**Published:** 2024-07-15

**Authors:** Wen-Jou Chang, Maria S. Baker, Eleonora Laritsky, Chathura J. Gunasekara, Uditha Maduranga, Justine C. Galliou, Joseph W. McFadden, Jessica R. Waltemyer, Bruce Berggren-Thomas, Brianna N. Tate, Hanxue Zhang, Benjamin D. Rosen, Curtis P. Van Tassell, George E. Liu, Cristian Coarfa, Yi Athena Ren, Robert A. Waterland

**Affiliations:** 1 Department of Pediatrics, USDA/ARS Children’s Nutrition Research Center, Baylor College of Medicine, Houston, TX United States; 2 Department of Animal Science, Cornell University, Ithaca, NY United States; 3 Animal Genomics and Improvement Laboratory, Agricultural Research Service, USDA, Beltsville, MD United States; 4 Department of Molecular and Cellular Biology, Baylor College of Medicine, Houston, TX United States; 5 Dan L Duncan Comprehensive Cancer Center, Baylor College of Medicine, Houston, TX United States; 6 Department of Molecular & Human Genetics, Baylor College of Medicine, Houston, TX United States

**Keywords:** Cow, Bovine, DoHAD, Systemic interindividual epigenetic variation, CoRSIV

## Abstract

**Background:**

We recently identified ~ 10,000 correlated regions of systemic interindividual epigenetic variation (CoRSIVs) in the human genome. These methylation variants are amenable to population studies, as DNA methylation measurements in blood provide information on epigenetic regulation throughout the body. Moreover, establishment of DNA methylation at human CoRSIVs is labile to periconceptional influences such as nutrition. Here, we analyze publicly available whole-genome bisulfite sequencing data on multiple tissues of each of two Holstein cows to determine whether CoRSIVs exist in cattle.

**Results:**

Focusing on genomic blocks with ≥ 5 CpGs and a systemic interindividual variation index of at least 20, our approach identifies 217 cattle CoRSIVs, a subset of which we independently validate by bisulfite pyrosequencing. Similar to human CoRSIVs, those in cattle are strongly associated with genetic variation. Also as in humans, we show that establishment of DNA methylation at cattle CoRSIVs is particularly sensitive to early embryonic environment, in the context of embryo culture during assisted reproduction.

**Conclusions:**

Our data indicate that CoRSIVs exist in cattle, as in humans, suggesting these systemic epigenetic variants may be common to mammals in general. To the extent that individual epigenetic variation at cattle CoRSIVs affects phenotypic outcomes, assessment of CoRSIV methylation at birth may become an important tool for optimizing agriculturally important traits. Moreover, adjusting embryo culture conditions during assisted reproduction may provide opportunities to tailor agricultural outcomes by engineering CoRSIV methylation profiles.

**Supplementary Information:**

The online version contains supplementary material available at 10.1186/s13059-024-03307-6.

## Background

Aiming to boost production efficiency, the cattle industry employs extensive breeding and selection techniques to enrich for desirable phenotypic traits. The advancement of breeding and selection processes has a long history, spanning over 100 years of data collection and prediction of genetic merit [1]. For example, the lifetime net merit index was devised to measure the expected lifetime net profit of dairy animals: it considers a wide array of traits (39 individual traits for Holstein cattle so far) and their associated predicted genetic and genomic merits, then ranks dairy animals with respect to economic gains [2]. In addition, compared to traditional methods, single-nucleotide polymorphism (SNP)-based genomic selection programs generate more than 100% increases in genetic improvements and yield [3].

Nonetheless, heritability estimates for production traits, linear conformation traits, and health and reproductive traits in Holstein cattle range from 0.01 to 0.60 [4]. Epigenetic marks stably affect gene expression [5]. So, similar to genetic variants, interindividual epigenetic variants could influence agriculturally important phenotypes. DNA methylation, which occurs in mammals mainly at CpG dinucleotides, is an epigenetic mechanism essential for differentiation and cell type-specific gene expression [6]. Moreover, as the most stable epigenetic mark [7], CpG methylation is a logical focus for studies trying to understand how individual epigenetic variation influences agriculturally important phenotypic outcomes. This has led, over the last decade, to increasing interest in understanding the causes and consequences of interindividual epigenetic variation in cattle [8]. Extensive research explores how variation in methylation among different tissues influences the expression of genes associated with agriculturally significant traits and evaluates effects of early nutrition and other environmental factors on DNA methylation [8].

Our studies in humans demonstrate that interindividual DNA methylation variants that are systemic (i.e. not tissue-specific) have particular utility in population epigenetics, as DNA methylation measurements in easily sampled tissues like peripheral blood provide information about epigenetic regulation throughout the body. Such epigenetic variation is called systemic interindividual variation (SIV). The first such variants identified in mammals were the mouse metastable epialleles *agouti viable yellow* and *axin-fused*. Early-embryonic establishment of DNA methylation at these loci occurs stochastically in the early embryo (i.e., not due to genetic variation) and was shown to be labile to maternal nutrition before and during pregnancy [9, 10]. Systemic interindividual epigenetic variants were subsequently identified in humans. Similar to mouse metastable epialleles, establishment of methylation at these loci is sensitive to periconceptional nutrition [11, 12] and other exposures including assisted reproductive technology [13].

The largest screen for systemic interindividual variants thus far identified nearly 10,000 correlated regions of systemic interindividual epigenetic variation (CoRSIVs) in the human genome [14] and showed, again, that these regions are particularly sensitive to periconceptional environment. Human CoRSIVs are under strong genetic control and tend to be located in genomic regions exhibiting long-range enrichments in LINE1 and LTRs and depletions of SINE transposable elements [15]. Despite growing interest in using DNA methylation to predict phenotypic outcomes in agricultural animals [8], CoRSIVs have not previously been identified in cattle.

The standard approach for identifying CoRSIVs is to perform DNA methylation profiling across multiple tissues (representing different embryonic germ layers) from each of multiple individuals [14]. Zhou et al. recently reported using whole genome bisulfite sequencing (WGBS) to profile DNA methylation in various tissues from each of two Holstein cows [16]. Here, by reanalyzing the data of Zhou et al. [16], we screened for cattle CoRSIVs. At a subset of regions, systemic interindividual variation was validated by bisulfite pyrosequencing in an independent sample of Holstein cattle (males and females). We also evaluated major hallmarks of human CoRSIVs: association with genetic variation and sensitivity to periconceptional environment.

## Results

### Genome-wide screen for cattle CoRSIVs

**Fig. 1:**
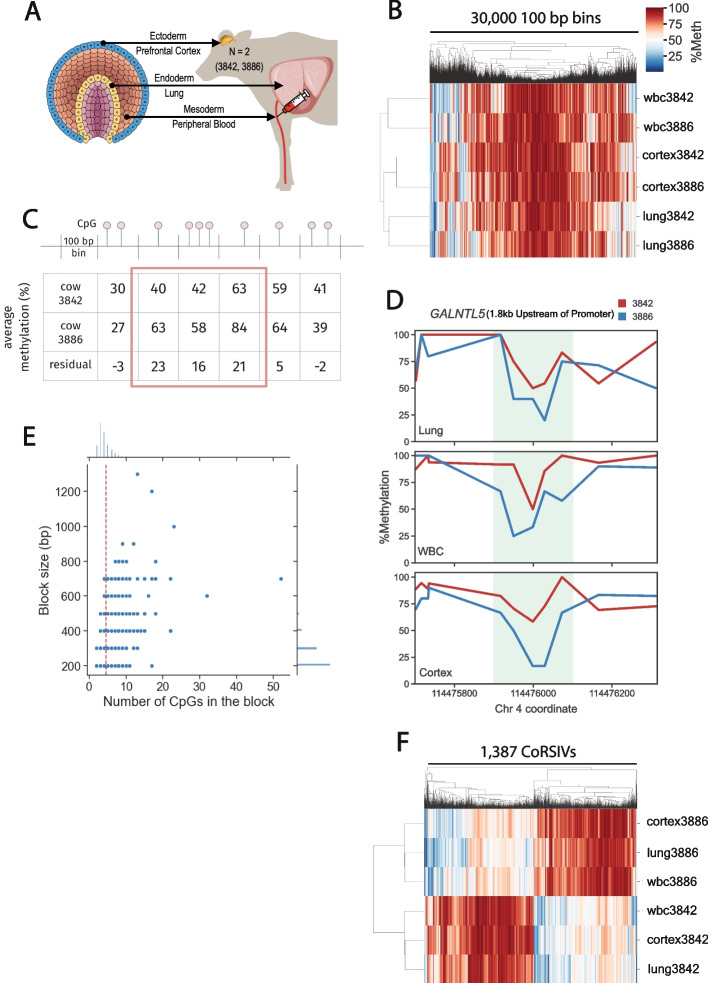
Screen for correlated regions of systemic interindividual variation (CoRSIVs) in cattle. **A** We analyzed WGBS data from three tissues representing the three embryonic germ layers from each of 2 cows (3842 and 3886; 6 methylomes total). **B** Unsupervised hierarchical clustering of average methylation groups the libraries by tissue (a random sample of 30,000 genomic bins is shown). **C** Illustration of our two-step approach: an example of a 300 bp block with concordant positive residuals is indicated by the red box. **D** A 200 bp CoRSIV (shaded region; 5 CpG sites) 1.8 kb upstream of the *GALNTL5* transcription start site illustrates consistent interindividual difference in all three tissues. **E** Scatter plot of number of CpGs per CoRSIV vs. CoRSIV size for all 1,263 CoRSIVs initially identified (following masking of CpG-SNPs). Subsequent analyses and the rest of this paper focus on the 217 CoRSIVs, each of which contains at least 5 CpGs (right of dotted line). **F** Unlike genome-wide bins, hierarchical clustering of average methylation in the 217 CoRSIVs groups the libraries by individual

We downloaded publicly available WGBS data on tissues from three embryonic germ layers: lung (endoderm), peripheral blood leukocytes (mesoderm), and frontal cortex (ectoderm) from each of two Holstein cows (one lactating and one dry (not lactating)) [16] (Fig. 1A). Due to the relatively low sequencing depth of these WGBS libraries (average coverage across all six libraries is ~ 18x (Additional file 1: Table S1), we began by imputing CpG methylation states on individual sequencing reads by Precise Read-Level Imputation of Methylation (PReLIM) to improve coverage [17]. We then partitioned the Bos taurus genome (bosTau9) into 100 bp bins and annotated all such bins containing at least one CpG site (hereafter referred to as ‘bins’).

Of the ~ 6 M bins with adequate coverage in all 6 libraries (see Methods), unsupervised hierarchical clustering of average CpG methylation in a randomly selected subset of 30,000 grouped the libraries by tissue (Fig. 1B). As in our previous screen in humans [14], to maximize genomic coverage we used a two-step analytical approach. First, we calculated an individual-level average methylation residual for each bin (%meth_cow3886_ – %meth_cow3842_). We then identified genomic blocks comprised of multiple consecutive bins with residuals ≥ 10%, in the same direction (Fig. 1C). Average methylation differences across all such blocks were balanced, showing no bias between the two cows (Additional file 2: Fig. S1A). In the second step, for all blocks we calculated a simplified systemic interindividual variation index (SIVI) [12]; a high SIVI indicates interindividual methylation differences that are similar within all three tissues.

Applying the same cutoffs as in our previous study (SIVI ≥ 20; 5 or more CpGs per CoRSIV) [12, 14] identified 1,387 candidate CoRSIVs (Additional file 1: Table S2). To evaluate potential effects of genetic variation on our screen, we utilized genotyping data of the two cows in the screen [16] to search for discordant SNPs at CpG sites (CpG-SNPs) within each of the 1,387 candidate CoRSIVs. Indeed, we identified a median of 2 CpG-SNPs per candidate CoRSIV (Additional file 2: Fig. S2). To guard against such genetic variation masquerading as epigenetic variation, we repeated both steps of the CoRSIV screen after masking all CpG-SNPs between the two cows. Applying a SIVI cutoff of  ≥ 20 [12] yielded 1,263 candidate CoRSIVs (Additional file 1: Table S3). An example of one at the promoter of *GALNT5* is shown in Fig. 1D. Across the 1,263 candidate CoRSIVs, average methylation differences were balanced across the two cows (Additional file 2: Fig. S1B). Also, the median number of CpGs per block was 3, and the median block size was 200 bp (Fig. 1E). Permutation testing (Additional file 2: Fig. S3) showed that less than 1.3% of all blocks satisfy the SIVI ≥ 20 criterion by chance; hence, each of the 1,263 CoRSIVs is statistically significant. However, many include only a few CpGs (Fig. 1E). To prioritize the most robust regions and consistent with our previous studies [12, 14], we focused all subsequent analyses on the 217 CoRSIVs containing at least 5 CpGs (Additional file 1: Table S4). Interestingly, across these 217 CoRSIVs the methylation differences were biased, reflecting higher average methylation in cow 3842 (Additional file 2: Fig. S1C; P = 2.12 × 10^–5^). Consistent with their systemic nature, unsupervised hierarchical clustering of average CpG methylation at these 217 CoRSIVs grouped the libraries by individual (Fig. 1F).

### Independent validation confirms systemic interindividual variation in DNA methylation

**Fig. 2:**
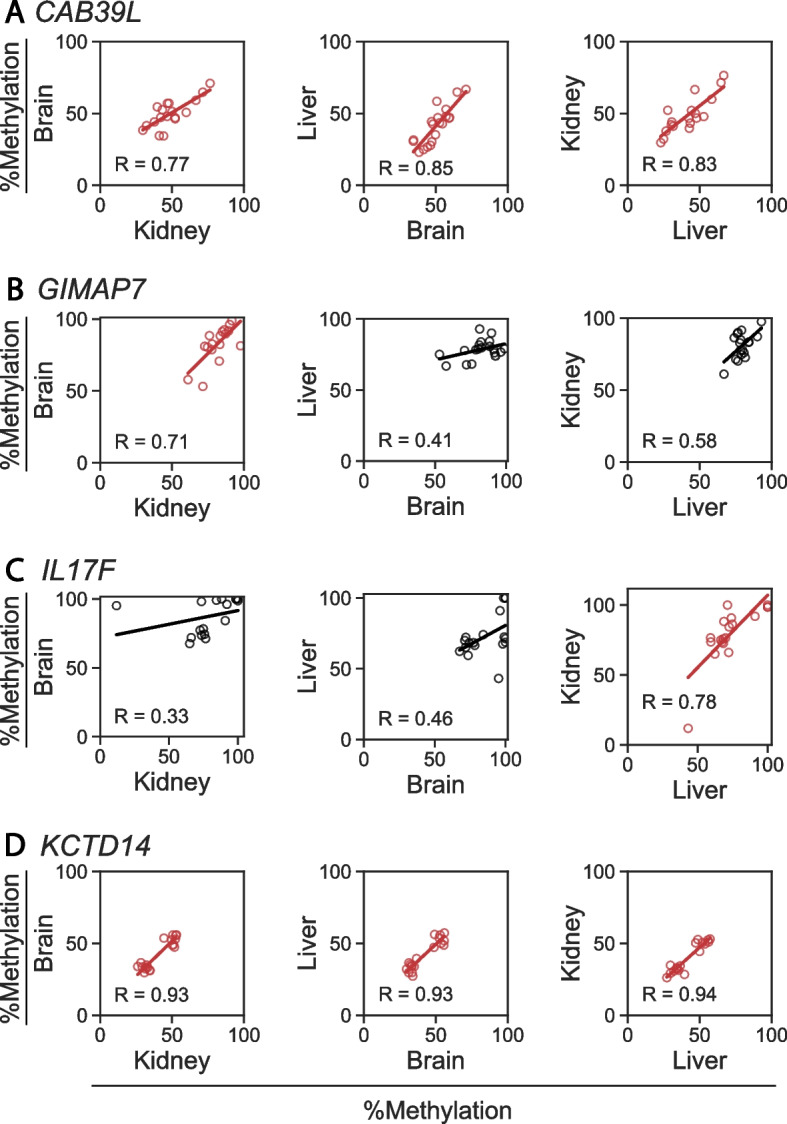
Independent validation of systemic interindividual variation at CoRSIVs we identified. Each scatter plot shows the inter-tissue correlation in DNA methylation based on quantitative bisulfite pyrosequencing in each of 20 Holstein calves (10 male, 10 female). **A** *CAB39L*, **B** *GIMAP7*, **C** *IL17F*, **D** *KCTD14.* Inter-tissue correlations with R ≥ 0.71 (the cutoff for validation) are highlighted in red

We obtained liver, kidney, and cerebral cortex from each of 20 Holstein calves and selected 11 of the 217 CoRSIVs for validation by pyrosequencing, as in our previous studies [11, 12] (Fig. 2; Additional file 1: Table S5). Positive validation results are shown in Fig. 2. Employing our standard criterion [12, 14], regions with r ≥ 0.71 (r^2^ ≥ 0.5) in at least one of the three inter-tissue correlations were considered validated. Four of 11 regions were validated, resulting in an overall validation rate of 36%. Of the two that showed high inter-tissue correlations in all three comparisons amongst liver, kidney, and brain (Fig. 2), methylation in each of these tissues was also correlated with that in peripheral white blood cells (Additional file 2: Fig. S4), indicating that methylation measurements in blood provide a proxy for epigenetic regulation throughout the body, as at human CoRSIVs [15].

### Cattle CoRSIVs are associated with genetic variation

**Fig. 3:**
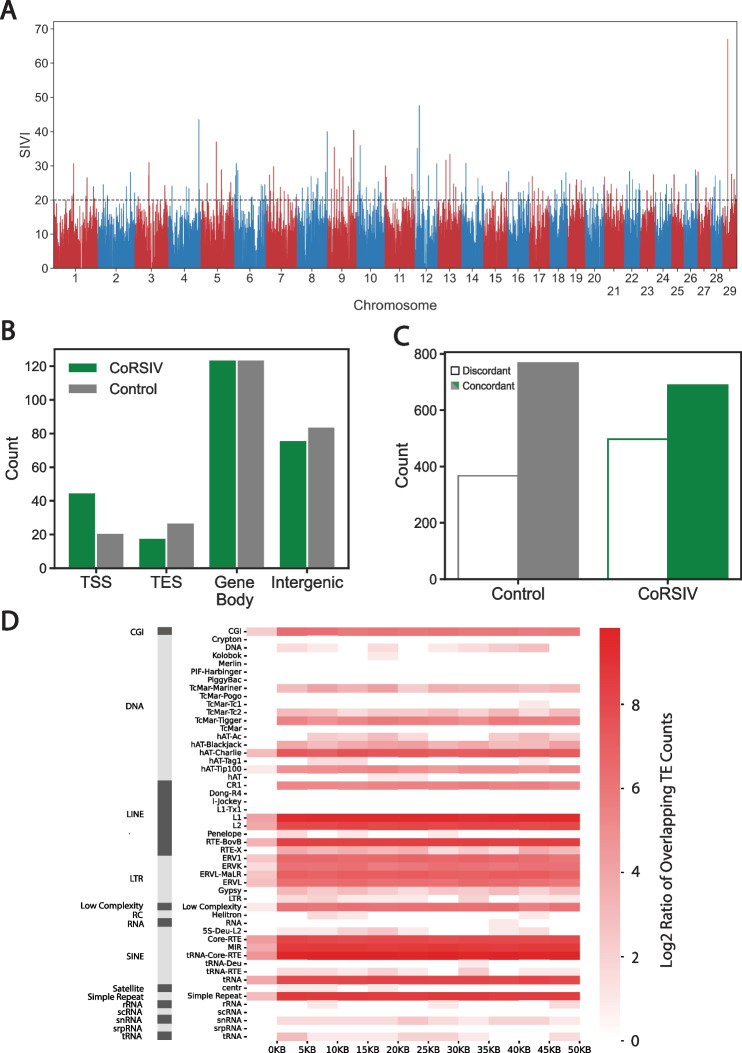
*Cattle CoRSIVs are enriched at transcription start sites and associated with genetic variation.* **A** Manhattan plot of systemic interindividual variation index (SIVI) for all genomic blocks with ≥ 5 CpGs. Blocks with SIVI ≥ 20 (dashed line) are considered CoRSIVs. **B** The overlap of CoRSIVs / controls with transcription start sites (TSS), transcription end site (TES), gene body, and intergenic regions. CoRSIVs are enriched at TSS. **C** The overlap with genetic variants in 1 kb windows centered on CoRSIVs / control regions. **D** CoRSIV-flanking regions (± 50 kb at 5 kb increments) show overlap with various classes of transposable elements, specifically LINE-1 and LTR elements. Column to the left of 0 KB mark shows direct overlap of CoRSIVs with transposable elements

As in humans, the 217 cattle CoRSIVs are non-uniformly distributed throughout the genome. A Manhattan plot (Fig. 3A) shows several chromosomes with regions of high and low CoRSIV density, such as chromosomes 9 and 3, respectively. To evaluate genomic characteristics of CoRSIVs, we generated a set of randomly selected control regions, each matched to one of the 217 CoRSIVs by chromosome, size, and CpG density (Additional file 1: Table S6, Additional file 2: Fig. S5). We used ShinyGO [18] to evaluate whether CoRSIVs are enriched for gene ontology terms related to biological process, cellular component, or molecular function, and found no significant enrichment. Relative to control regions, cattle CoRSIVs were enriched at transcription start sites (Fig. 3B; P = 2.1 × 10^–3^). No significant differences were found between CoRSIVs and controls relative to gene bodies, transcription end sites (TES), or intergenic regions (Fig. 3B).

Most human CoRSIVs are associated with genetic variants [15]. We therefore analyzed genotyping data of Zhou et al. [16], including both SNPs and insertions/deletions (indels), on the two cows in our screen to investigate genetic variation in 1 kb windows centered on CoRSIV and control regions. Indeed, whereas genetic variants in the vicinity of control regions were over 100% more likely to be concordant than discordant, in the vicinity of CoRSIVs this increment was just 38% (Fig. 3C; P = 2.3 × 10^–6^). In humans, genomic regions flanking CoRSIVs show long-range enrichment of specific classes of LINE1 and LTR transposable elements, and depletion of CpG islands (CGIs) and SINEs [15]. Possibly due to the limited number of CoRSIVs identified here, we detected no long-range enrichment of transposable elements at genomic regions flanking cattle CoRSIVs. Although there are many LINE1, LINE2, and LTR elements within 50 kb of cattle CoRSIVs (Fig. 3D), the patterns of their distributions were not obviously different from those in the vicinity of control regions (Additional file 2: Fig. S6).

### Establishment of DNA methylation at cattle CoRSIVs is sensitive to periconceptional environment

**Fig. 4:**
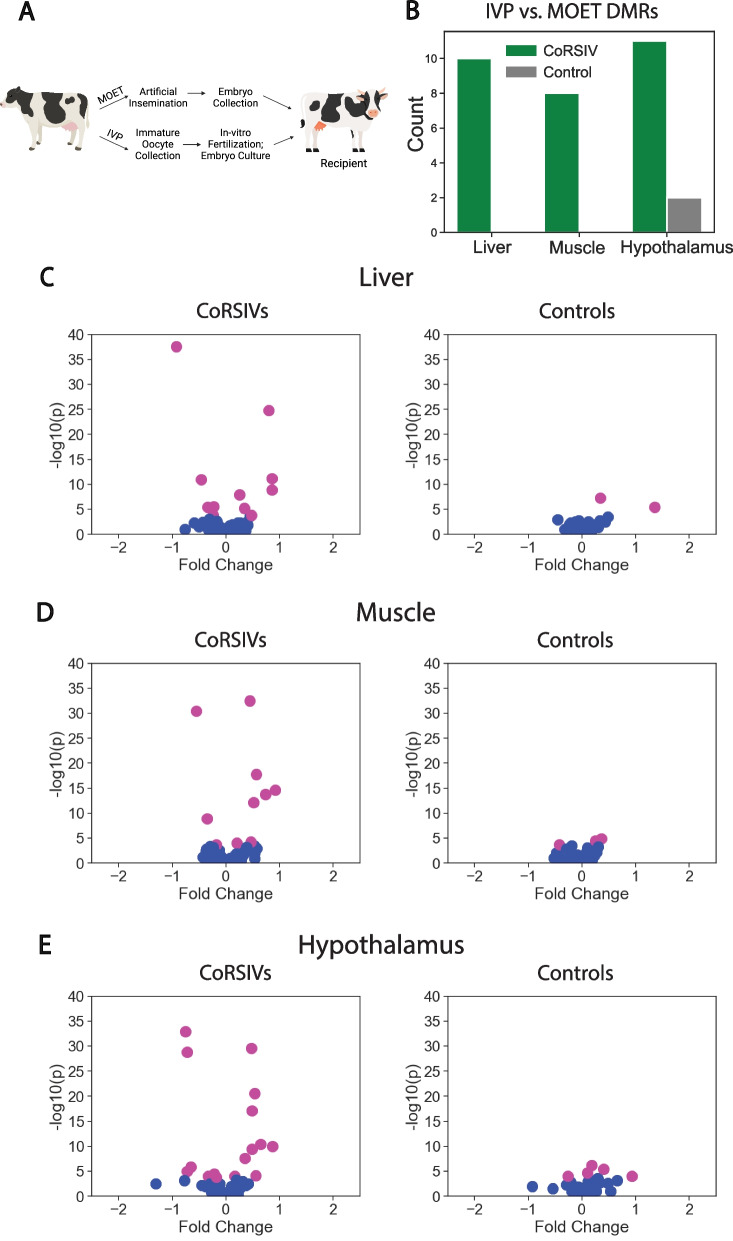
*Establishment of DNA methylation at cattle CoRSIVs is particularly labile to periconceptional environment.* **A** Graphic illustration of IVP/MOET experiments. **B** Overlap of differentially methylated regions (DMRs) between IVP and MOET offspring with CoRSIVs and control regions in three tissues: liver, muscle, and hypothalamus. **C-E** For the IVP vs. MOET experiments, volcano plots illustrating significant differences in average methylation between IVP and MOET at 217 CoRSIVs and matched control regions in **(C)** liver, **(D)** muscle, and **(E)** hypothalamus. Each point represents a CoRSIV or control region. P values are adjusted for multiple testing; significant p values are shown in pink

Embryo transfer is one of the most common forms of assisted reproductive technologies (ART) used for cattle reproduction, involving the creation of embryos either through in vivo methods (multiple ovulation and embryo transfer (MOET)) or through in vitro embryo production (IVP), in which immature oocytes are collected from live cows and fertilized ex vivo*,* followed by in vitro embryo culture for 5–7 days prior to implantation in a recipient cow [19] (Fig. 4A). We used an existing WGBS data set [19] to test whether DNA methylation at CoRSIVs is particularly sensitive to the effects of embryo culture during IVP.

Rabaglino et al. [19] obtained multiple tissues from three-month-old calves produced by IVP (n = 4) and by MOET (control, n = 4) and performed WGBS. We analyzed the WGBS data on liver, muscle, and hypothalamus (representing embryonic endoderm, mesoderm, and ectoderm, respectively) and identified differentially methylated regions (DMRs) between IVP and MOET calves (Additional file 1: Table S7-S9). Our statistical tests were based on comparing CoRSIVs with matched control regions. Given the small number of animals studied (n = 4 per group), we recognized that if there are more SNPs affecting CpG sites (CpG-SNPs) within CoRSIVs than control regions, chance genetic differences between the groups could masquerade as methylation differences. Using data from the 1000 Bull Genomes Project [20], we found nearly the same number of CpG-SNPs at CoRSIVs and control regions (86 and 76, respectively). Moreover, of the 217 CoRSIVs or control regions, nearly the same number contain at least one CpG-SNP (58 and 59, respectively), alleviating concerns of potential genetic confounding. Strikingly, DMRs between the IVP and MOET calves were over five to ten times more likely to overlap CoRSIVs than control regions, depending on the tissue (Fig. 4B; P = 4.4 × 10^–3^, 0.01, 0.03 for liver, muscle, and hypothalamus, respectively; Additional file 1: Table S10-S12). Generally, the effects of IVP on DNA methylation varied by tissue. Across all DMRs, compared to the MOET group, IVP animals showed a bias toward lower methylation in liver (Additional file 2: Fig. S7A; P = 1.48 × 10^−31^), and higher methylation in hypothalamus (Additional file 2: Fig. S7B; P = 1.32 × 10^−82^) and muscle (Additional file 2: Fig. S7C; P = 4.41 × 10^−10^). Conversely, consistent with their systemic nature, this tissue-specificity was not found at DMRs overlapping CoRSIVs, which tended to exhibit higher methylation in IVP cattle in all three tissues (Additional file 2: Fig. S8). Because of the relatively small number of DMRs, we conducted a complementary analysis using chi-square tests based on read-level counts of methylation in the IVP vs. MOET offspring. This analysis detected many more group differences in methylation at CoRSIVs than in control regions (Fig. 4C-E; P = 0.024, 0.09, 0.025 for liver, muscle, and hypothalamus), corroborating the DMR-based results. A chi-square test across the three tissues yielded a P value of 0.025.

## Discussion

Here, we show for the first time that CoRSIVs, first identified in humans [14], also exist in cattle. Moreover, cattle CoRSIVs share major characteristics with human CoRSIVs. First, both cattle and human CoRSIVs are strongly associated with genetic variation. Our latest analysis of human CoRSIVs identified over 70-times more methylation quantitative trait loci (mQTL) than previously detected [15]. Although, with only two individuals, our study was not powered to quantify mQTL, our results show that relative to control regions, cattle CoRSIVs are more likely to be associated with cis genetic variation. In this regard, it is surprising that across the 217 regions we identified, methylation tended to be higher in one cow than the other (Additional file 2: Fig. S1C). This could be attributable, however, to either differences in periconceptional environment or a trans genetic effect that generally promotes DNA methylation during early embryonic development.

Just like at murine metastable epialleles [9, 10] and human CoRSIVs [14], our analyses of cattle produced by IVP vs. MOET demonstrate that establishment of DNA methylation at cattle CoRSIVs is particularly sensitive to periconceptional environment. This finding indicates that genomic regions showing systemic interindividual variation in DNA methylation are generally labile to early embryonic environment. In this regard, our results are contrary to those of a recent study in mice [21] which concluded that regions of systemic interindividual variation (SIV) do not generally exhibit such plasticity. It is important to note that unlike mouse metastable epialleles such as *agouti viable yellow* and *axin-fused*, which are generally studied within inbred populations, at CoRSIVs in outbred mammalian populations establishment of DNA methylation is influenced both by periconceptional environment and genetic variation [11–15]. There does not appear to be a dichotomization of CoRSIVs that are influenced by early environment and those associated with genetic variation.

Despite these strengths, our study is not without weakness. First, only Holstein cattle were included. Given the strong influence of genetics on CoRSIVs [15], it is likely that some CoRSIVs will be breed-specific. In addition, WGBS data on only two cows were available for this screen, limiting our ability to both broadly detect interindividual variants and characterize genetic influences at these loci. Notably, although we detected only 217 CoRSIVs in this screen, this number is comparable to the 109 SIV regions we detected in our first unbiased WGBS screen in humans, which was also based on just two individuals [12]. In addition, the WGBS datasets we used were not very deep, compromising the genomic coverage of the screen. Likely due to this limited sample size and sequencing depth, the overall validation rate was somewhat lower than in our human studies [14]. Future studies profiling genomic DNA methylation in tissues representing all three embryonic germ layer lineages from a larger number of individuals will be needed to better understand systemic interindividual epigenetic variation in cattle.

Nonetheless, we envision great potential for CoRSIVs to be useful in predicting and tailoring phenotypic outcomes that boost production efficiency in cattle, particularly given that CoRSIV methylation in peripheral blood can provide information about epigenetic regulation throughout the body (Additional file 2: Fig. S4). Human studies have shown that methylation at CoRSIVs is stable for years in the same individual [12, 22, 23]. It is possible, therefore, that profiling CoRSIV methylation in peripheral blood DNA of calves could someday be utilized to identify those individuals most likely to exhibit traits favorably associated with productivity in adulthood.

Assisted reproductive technologies (ART) such as artificial insemination (AI), embryo transfer (ET), and in vitro fertilization (IVF) are commonly used in cattle production. In combination with genomic selection and selective breeding, ART allows for maximizing and accelerating genetic gain from cattle with superior genetics, leading to improved cattle production efficiency and sustainability [24, 25]. In North America, the adoption of AI for cattle breeding is prevalent among nearly 80% of dairy producers, and 4% of beef producers [26]. In addition to genetic profiling, we have identified an additional level of individuality at the epigenetic level. Consideration of epigenetic variation at CoRSIVs may provide a useful complement to management strategies based on genotyping [3]. We are not proposing that CoRSIV methylation is transgenerationally heritable, but that it may be a useful variable for optimizing traits within each generation. For example, a potential application for CoRSIVs is to address low female fertility in dairy cattle, by characterizing differences in CoRSIV methylation between individuals with optimal vs. poor fertility. This knowledge could subsequently be used to inform selective breeding practices. During the weaning stage, for example, calves could undergo methylation profiling at CoRSIVs, enabling the identification of female calves with profiles indicative of superior fertility. Selecting such individuals for further breeding could potentially improve overall reproductive efficiency within dairy cattle populations, promoting sustainable dairy farming. In support of this possibility, in a rat model, *GIMAP7* (Fig. 2B) was recently shown to promote oxidative stress and apoptosis in ovarian granulosa cells [27]. Beyond such selection for desirable traits within each generation, our results on periconceptional influence, specifically embryo culture during IVP, suggest the possibility of manipulating cow nutrition or the composition of culture media used in IVP to epigenetically optimize phenotypic outcomes [28, 29].

## Conclusion

In this study, we have shown that CoRSIVs exist in the cattle genome. However, the data in this project are limited by low coverage and an insufficient number of individuals. For future studies, larger numbers of animals and deep WGBS in different tissues will be needed to better identify and characterize cattle CoRSIVs. Given the strong influence of genetics on CoRSIVs [15], investigating CoRSIVs in multiple cattle breeds will also be important. CoRSIVs may one day prove useful for boosting production efficiency and profitability in dairy and beef cattle production systems by providing additional metrics for selection and culling strategies and opportunities for epigenetic engineering based on embryo culture during assisted reproductive technologies. More broadly, by suggesting that CoRSIVs may be a feature of mammalian genomes in general, our results indicate that such opportunities for agricultural improvement are not limited to cattle.

## Materials and methods

### Publicly available datasets used

Zhou et al. generated WGBS data for 13 different tissues in Holstein cattle [16] (GEO accession number: GSE147087). We selected 2 individuals with data available for 3 tissues (cortex, lung, white blood cell) in our analysis. Cow 3842 was approximately 3.5 years old and lactating, while cow 3886 was approximately 3.0 years old and was dry, not lactating.

Rabaglino et al. [19] obtained samples from 6 tissues from three-month-old calves produced by IVP (n = 4) and by MOET (control, n = 4) and performed WGBS (GEO accession number: GSE223098). We utilized 24 samples (8 calves, 3 tissues each (liver, muscle, hypothalamus)) in our analysis.

Run8 and Run9 of the 1000 Bull Genomes Project [20] are deposited in the European Nucleotide Archive under accession number PRJEB42783 and PRJEB56689, respectively. We combined the two runs and included only 254 Holstein samples. We only included variants with valid GT in at least 200 Holstein samples and minor allele frequency ≥ 0.05 in all subsequent analysis. We used Bcftools 1.19 in handling VCF files.

### WGBS data processing

We performed quality control checks, alignment, and methylation calling as previously described [14]. We aligned pair-ended sequencing reads to the ARS-UCD1.2 (bosTau9) bovine reference genome. Lastly, we removed duplicated alignments using deduplicate_bismark from Bismark.

### CoRSIV screen

To increase the relatively low coverage of the existing data (~ 18x), we first ran Precise Read-Level Imputation of Methylation (PReLIM) [17], which imputes CpG methylation states on individual sequencing reads, on the coverage file generated by Bismark tool methylation extractor.

Genetic variation at CpG sites can affect apparent methylation level. For example, if a C  to T SNP occurs at the cytosine in a CpG site, such cytosine would be misrepresented as an unmethylated cytosine in subsequent WGBS sequencing. Hence, genetic variations can create artifactual methylation differences between the two individuals in the screen. Using genotyping data published by Zhou et al. on the lung tissue of the two Holstein cows used initially in the screen, we identified 842,724 discordant SNPs affecting either a C or G in a CpG site with quality score ≥ 30, which were subsequently removed from the coverage file and all subsequent methylation analysis. We then adopted a two-step analytical approach to maximize genomic coverage.

### First-step

We partitioned the Bos taurus genome (bosTau9) into 100 bp bins and annotated all such bins containing at least one CpG site. We focus on the ~ 10.5 M 100 bp bins with adequate coverage (roughly 83% of all ~ 12.6 M 100 bp bins containing at least one CpG) in at least two tissues in each animal. For a bin with n CpGs, if n ≤ 2, the bin is adequately covered when all CpGs are covered by 5 or more reads; if n > 2, the bin is sufficiently covered when at least (n/2, rounded up) CpGs are covered by 5 or more reads. Then, we calculated an average individual-level methylation residual (%meth cow_3886_ – %meth cow_3842_) for each bin, based only on tissues with adequate coverage.

### Second-step

We identified 138,192 genomic blocks comprised of two or more consecutive bins with residuals having absolute values ≥ 10%, in the same direction. For all such blocks, we calculated a systemic interindividual variation index (SIVI); a high SIVI reflects interindividual methylation differences that are similar within all three tissues.

Systemic Interindividual Variation Index (SIVI) formula:

$$SIVI\hspace{0.17em}=\hspace{0.17em}A\hspace{0.17em}+\hspace{0.17em}B$$

Where

$x$ = cortex residual (%meth cow_3886_—%meth cow_3842_)

$y$ = lung residual (%meth cow_3886_—%meth cow_3842_)

$z$ = wbc residual (%meth cow_3886_—%meth cow_3842_)

$$\text{A}\hspace{0.17em}=\hspace{0.17em}\sqrt[3]{\left|x\cdot y\cdot z\right|}$$

$$\begin{array}{cc}\text{B }= -sd(x, y, z)& (\text{sd}\hspace{0.17em}=\hspace{0.17em}\text{standard deviation})\end{array}$$

The A term rewards high interindividual differences in methylation. The B term rewards consistent interindividual differences in methylation percentages across all three tissues. The initial formula developed by Silver and Kessler et al. [12] included a C term rewarding consistent methylation percentages across all tissues, but we chose to drop this term based on observations that interindividual variation can still be highly correlated across tissues even when average % methylation values differ among tissues [15].

### Independent validation by bisulfite pyrosequencing

We obtained liver, kidney, cerebral cortex (representing endoderm, mesoderm, and ectoderm, respectively), and blood from each of 20 Holstein calves (10 females and 10 males, age 2 to 17 days). The sets of tissues representing the germ layers were chosen to be different in the validation experiment rather than in the initial screen, to better test the systemic nature of interindividual variation at CoRSIVs. The calves were housed with standard care at the Cornell University Ruminant Center (Harford, NY) and transported to the abattoir of Frank Morrison Hall (Ithaca, NY) on the day of tissue collection. Euthanasia was performed by penetrating captive bolt followed by exsanguination during which blood was collected. Immediately following euthanasia, different types of tissues were collected from each calf and snap frozen on dry ice. Quantitative bisulfite pyrosequencing was performed to evaluate inter-tissue correlations. When designing pyrosequencing assays, data on common SNPs in Holstein cattle in the 1000 Bull Genomes Project [20] were used to ensure that SNPs neither overlapped the PCR or sequencing primers, nor were within the sequence analyzed. To validate the sensitivity and quantitative accuracy of each pyrosequencing assay, we measured standards of known percentages of fully methylated and unmethylated Holstein genomic DNA [12, 30]. DNA isolation and bisulfite conversion were conducted similarly to our previous studies [11]. The primers used and assay validation data are included in Additional file 1: Table S5. High inter-tissue correlation (for example, liver vs. kidney) is a hallmark of SIV. Similar to our study in humans [12], a CoRSIV was considered validated if at least one of the three inter-tissue correlations (liver-kidney, kidney-cortex, liver-cortex) was at least 0.71 (r^2^ = 0.5). For validated CoRSIVs, we also tested respective correlation of three tissues with peripheral blood, to determine whether methylation status in peripheral blood can be used to infer methylation status in tissues throughout the body.

### Control regions

Control regions were identified in a similar fashion as in previous study [14], with a few changes. We started by removing CoRSIVs from the list of preprocessed 100 bp bins to avoid selecting CoRSIVs as controls. It is unlikely to identify the same control region for different CoRSIVs, so we sampled them with replacement. To find a matching control region, we first matched by chromosome, then prioritized matching CpG numbers, then genomic size. If the selected control didn’t have the exact same CpG density as its CoRSIV, then we made another attempt at selecting a control region. If after 1000 attempts the CpG density was still not achieved, we would increment target genomic size by 100, then reiterated the random selection process. At last, we ensured CoRSIVs and controls don’t overlap with each other, and control regions don’t overlap within themselves.

### Permutation test for CoRSIVs

We conducted a permutation test on all blocks to determine if CoRSIVs were statistically significant. The process generally follows that in our previous study [14]. We began with blocks that were identified after block-building criteria of delta ≥ 10 in the same direction and performed the following steps on each block for 1000 iterations:

1. Scramble library ID for all six libraries (2 subject × 3 tissues)
2. Compute SIVI for the current block based on permuted library ID

### Assessing genomic characteristics of CoRSIVs

#### Association with genic features

CoRSIVs / control regions within 2.5kp of a transcription start site (TSS) were categorized as TSS CoRSIVs / control regions. CoRSIVs / control regions within 2.5kp of a transcription end site (TES) were categorized as TES CoRSIVs / control regions. CoRSIVs / control regions overlapping genes were categorized as Gene Body CoRSIVs / control regions. CoRSIVs / control regions that did not satisfy any of the above requirements were categorized as intergenic CoRSIVs / control regions. One region may be classified into multiple categories, for example in cases when the TSS of one gene is proximal to the TES of another.

### Association with genetic variants

Examining both 1000 Bull SNPs and lung genotyping on the two cows provided by Zhou et al., we counted the number of overlapping discordant and concordant genetic variations (including both SNPs and indels) within 1 kb windows centered on CoRSIVs and control regions, respectively, using Bedtools v.23.0.

### Associations with transposable elements and CpG islands

The repeat definitions in the RepeatMasker track were obtained from the UCSC genome browser build ARS-UCD1.2 and then analyzed at the level of repeat class and family. Additionally, CpG islands defined by the UCSC genome browser were downloaded. We examined regions ± 50 kb of CoRSIVs and controls at 5 kb increments (± 0-5 kb, ± 5 kb-10 kb, etc.) and counted CoRSIVs and controls’ number of overlaps with each class of transposable elements.

### Investigating the effect of assisted reproductive technology on CoRSIVs

Using WGBS data generated by Rabaglino et al. [19], we called Bismark methylation extractor on BAM files and converted coverage files for both the IVP group and MOET group to the input format required for Dispersion Shrinkage for Sequencing data (DSS) [31] of four columns: chromosome number, genomic coordinate, total read counts, and number of methylated reads. They were then passed into DSS in the Bioconductor package of R to identify differentially methylated regions (DMRs) between the two groups, with the parameters p = 0.05 and delta = 0.05. We used Bedtools to determine overlaps between the resulting DMRs and CoRSIVs / control regions, respectively. An overlap was considered valid if the overlap was at least half the size of either the CoRSIV or control region or the DMR (whichever was smaller). Because of the relatively low number of DMR overlaps identified, as a complementary approach, we performed a read-level analysis to identify CoRSIVs or control regions showing statistically significant differences in methylation associated with IVP vs. MOET group. We performed chi-square goodness of fit tests on each CoRSIV or control region, using 2 × 2 contingency tables with rows designating the number of methylated and unmethylated reads for all CpGs in the CoRSIV or control region, and columns designating the IVP or MOET group. The chi-square tests were performed by chi2_contingency in scipy.stats of Python, with Yate’s correction turned on. The p-values were then adjusted for multiple testing. To obtain a p value for each of the three tissue and a p value for three tissues combined, we again performed chi-square goodness of fit tests with contingency tables recording the number of CoRSIVs / controls that show statistically significant differences in methylation level between the IVP and MOET groups, versus the number of CoRSIVs / controls that show no difference.

### Supplementary Information

Additional file1: Supplementary Tables S1-S12 with descriptions.

Additional file2: Supplementary Figures S1-S8 with legends.

Additional file3: Peer review history.

## Data Availability

All datasets used in this manuscript are publicly available, as described under Materials and Methods – Publicly available datasets used. Accession information is as follows: WGBS data: Zhou, Y., et al. GSE147087 [32] and Rabaglino, M.B., et al. GSE223098 [33]. SNV data: Hayes, B.J. and H.D. Daetwyler PRJEB42783 (Run8) [34], PRJEB56689 (Run 9) [35].
