# Supplementary material for: Systemic interindividual DNA methylation variants in cattle share major hallmarks with those in humans
Source: Genome Biol. 2024 Jul 15;25:185. doi: 10.1186/s13059-024-03307-6 (PMC11247883; doi:10.1186/s13059-024-03307-6)
Supplement: Supplementary file 2 — Additional file2: Supplementary Figures S1-S8 with legends. [file 13059_2024_3307_MOESM2_ESM.docx]

#
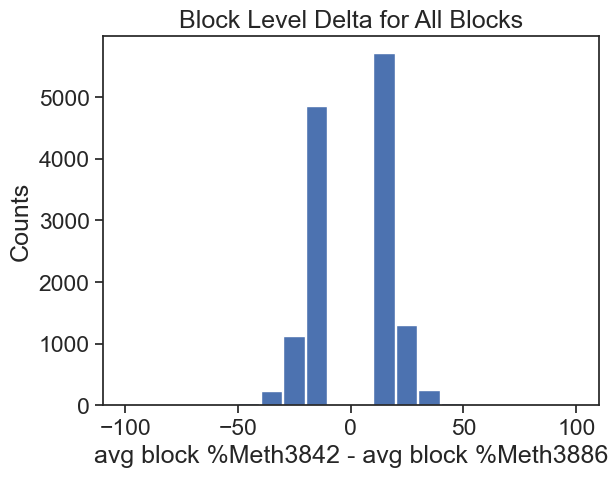
 (A)


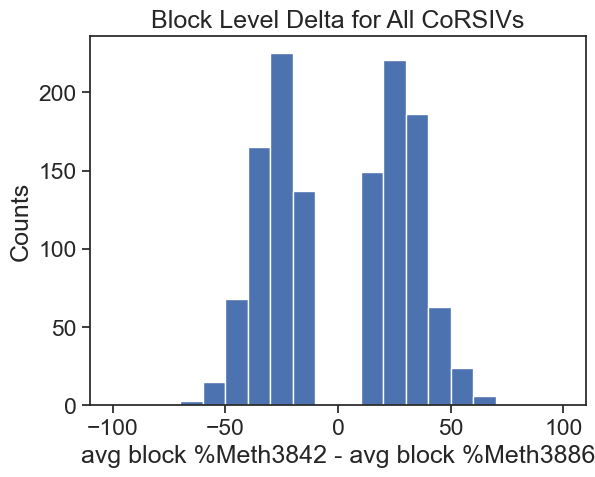
 (B)


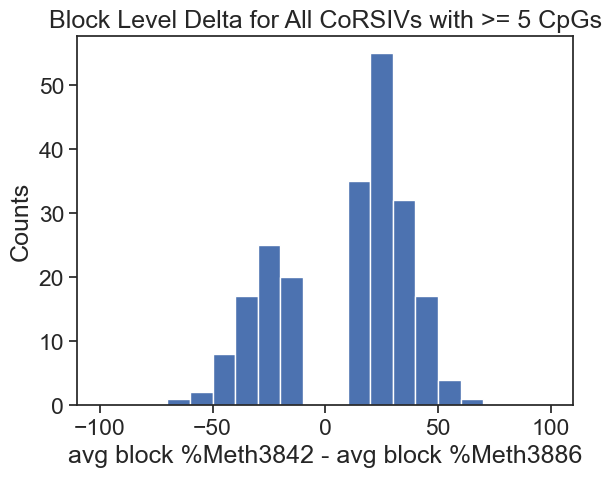


(C)

**Fig. S1:** Block level %methylation difference between the two cattle in the screen show equal methylation level **(A)** for all blocks, and **(B)** for all 1,263 CoRSIVs, but generally higher methylation level in cow 3842 **(C)** for all 217 CoRSIVs with 5 or more CpGs (P = 2.12 x 10^-5^).


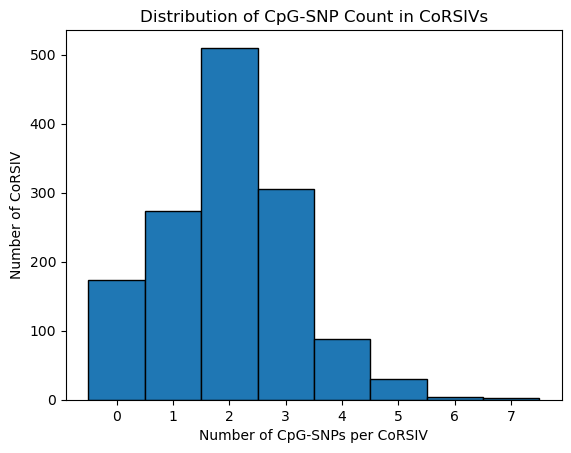


**Fig. S2: CpG-SNPs Distribution:** Distribution of number of SNPs that are discordant between the two cows at CpG sites (CpG-SNPs) in each of the 1,387 CoRSIV prior to SNP filtering. The median is 2.


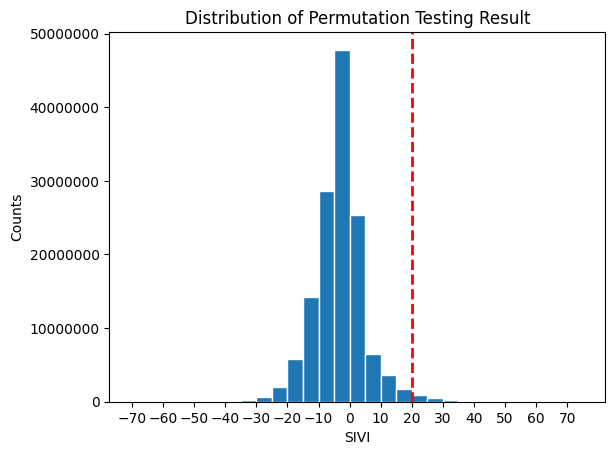


**Fig. S3: Permutation Testing Result:** Less than 1.3% of the blocks have a SIVI greater than or equal to 20.


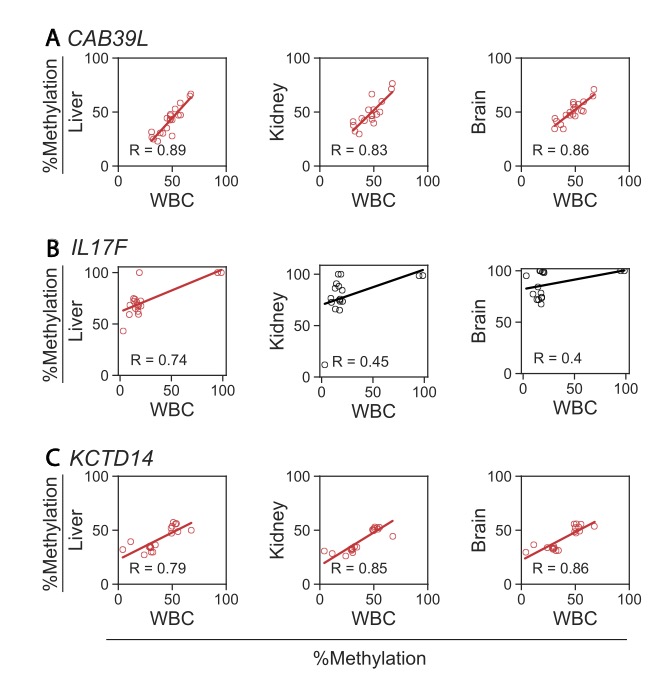


**Fig. S4: Additional validation result with respect to white blood cells:** Each row shows inter-tissue correlations in DNA methylation between liver, kidney, and brain vs. white blood cells (WBC). These are based on quantitative bisulfite pyrosequencing in each of 20 Holstein calves (10 male, 10 female). **(A)** *CAB39L*, **(B)** *IL17F*, **(C)** *KCTD14*. Inter-tissue correlations r ≥ 0.71 (our cutoff for validation) are highlighted in red.


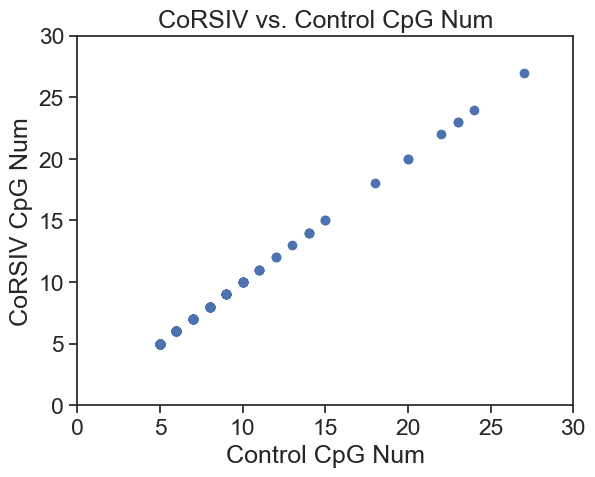


# (A)

(B)


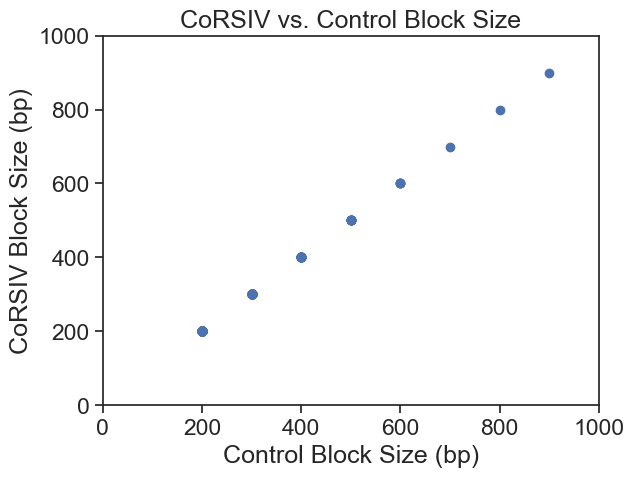


**Fig. S5: CoRSIV vs. Control Regions** **(A)** All control regions contain the same number of CpGs as their CoRSIV counterparts. **(B)** All control regions are the same in size as their CoRSIV counterparts.


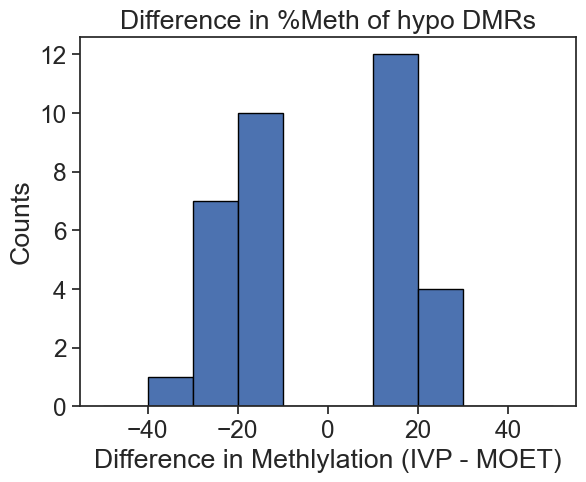

**Fig. S6: Regions flanking control regions (at +/-5kb increment, up to +/- 50kb) show overlap with various classes of transposable elements.** The column to the left of 0kb indicates direct overlap of control regions with transposable elements.


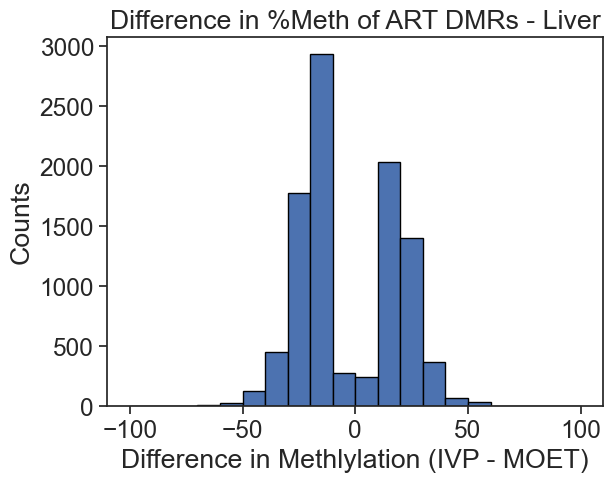


(A)


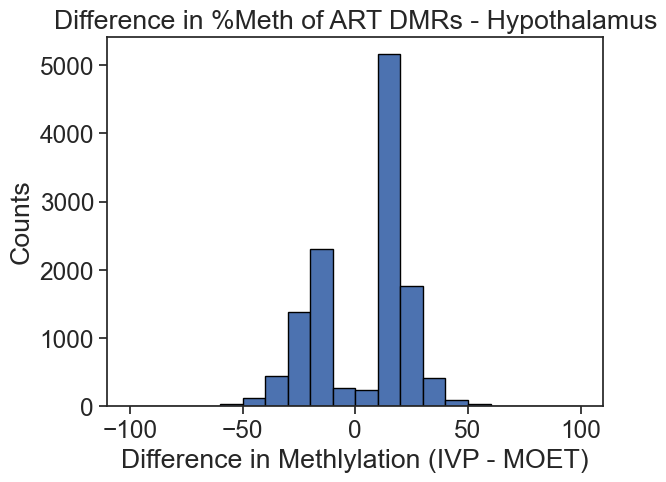


(B)


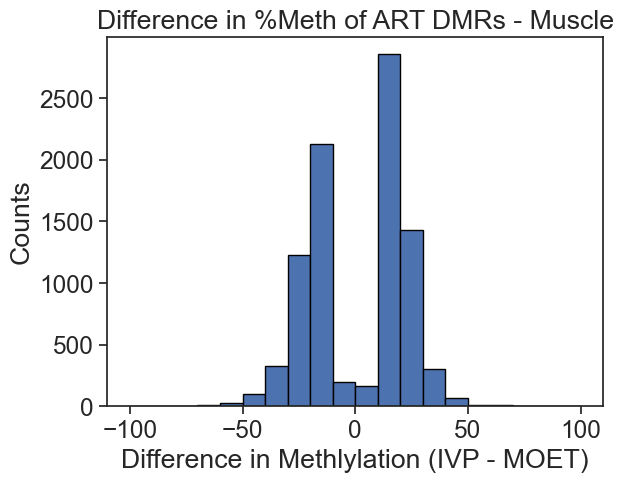


(C)

**Fig. S7: The entire set of IVP vs. MOET DMRs shows imbalance in different directions in different tissues: (A)** In liver, methylation is generally higher in the MOET group (P = 1.48 x 10^-31^), **(B)** In hypothalamus, methylation is generally higher in the IVP (P = 1.32 x 10 ^-82^), **(C)** In skeletal muscle, methylation is generally higher in the IVP group (P = 4.41 x 10 ^-10^).


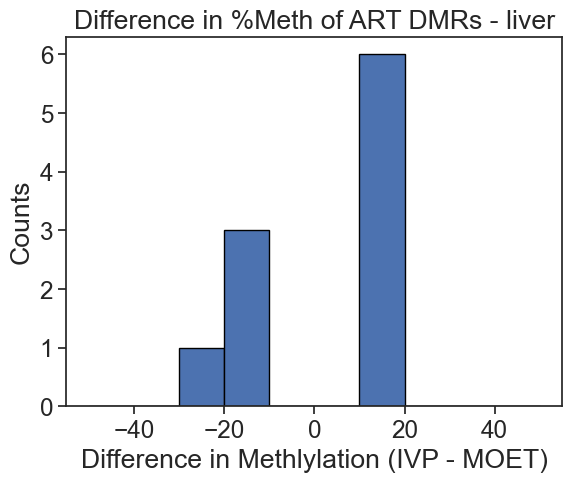
 (A)


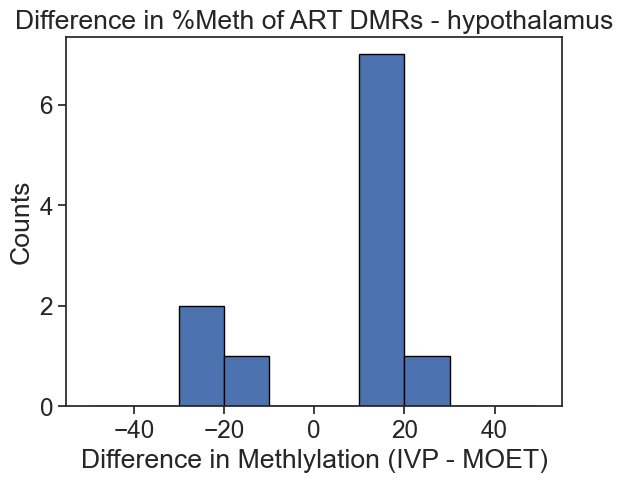
(B)


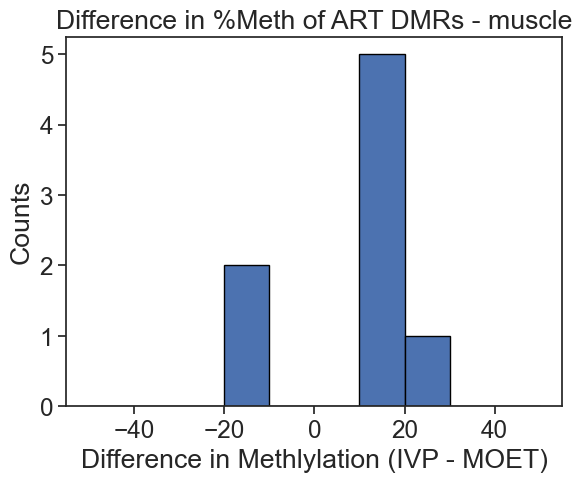
(C)

**Fig. S8: At IVP vs. MOET DMRs overlapping CoRSIVs, methylation is generally higher in the IVP** group in all three tissues: **(A)** liver, **(B)** hypothalamus, and **(C)** muscle. Perhaps due to the low sample size, however, these biases were not statistically significant.
