# Supplementary material for: Systemic interindividual DNA methylation variants in cattle share major hallmarks with those in humans
Source: Genome Biol. 2024 Jul 15;25:185. doi: 10.1186/s13059-024-03307-6 (PMC11247883; doi:10.1186/s13059-024-03307-6)
Supplement: Supplementary file 3 — Additional file3: Peer review history. [file 13059_2024_3307_MOESM3_ESM.docx]

**Review history**

**First round of review**

**Reviewer 1**

This is an superficial but interesting paper exploring the DNA methylation landscape in bovine in regard to different tissues differences and similarities. It is clear that it is important to document the variability at the cytosine methylation sites in the genome to better understand what seems to be inherited, genetic dependent or environment dependent.
It is a copy of a more extensive human study already published and has reduced value since no specific hypothesis or comparison of phenotypes is included.

The principal limitation of this paper is the number of animals used (n=2). And the number of tissues compared. (n=3) for the initial landscape analysis. This remains statistically valid but probably limits the number of true CORSIVS presented here compared to a large populated study.

The authors have been quite conservative to reduce false positive by using blocs with at least 5 CpGs, which probably represent the most functional methylation changes especially if in the promotors.

What is missing in this paper is the description of variable sites that are not related to genetic (SNVs) but potentially dependent on the environment and variable in all animals. This is where, having a population of > 12 ,would be useful as many of the methylation changes observed in defined environments ( food/temperature/IVF) are not related to sequences differences and are likely the ones that could provide additional information compared to genotyping

The methionine experiment is not well discussed in terms of what additional benefit it provides and why CoRSIVs have the same hypomethylation in methionine supplemented animals.

Specific comments

Abs: I would wait for e few more species before making this conclusion: suggesting these
systemic epigenetic variants may be common to all mammals


Line 119: Do you have evidence in bovine that the blood DNA methylation is a good proxie for the rest of the tissues ?

Line 265 For the IVP animals, since the methylation changes may be caused by early culture conditions, the CoRSIVs then reflect changes common to all IVP vs MOET and do not represent a genetic programming but potentially a genetic susceptibility to the culture conditions.


Line 206: a validation study (pyrosequencing or targeted PCR post. conversion) should be done to assess if methylation changes observed at these locus are present in more animals ( with different SNVs) for the following genes F2RL3, SIN3B, MACROD2, API5, and PKHD1.


Line 325: Please explain the following sentence or justify its value :
Hence, CoRSIVs provide an helpful tool when using early embryonic development to guide epigenetic outcome.

Line 333. This supposition has already been demonstrated and please provide references

1: Estrada-Cortés E, Ortiz W, Rabaglino MB, Block J, Rae O, Jannaman EA, Xiao Y,
Hansen PJ. Choline acts during preimplantation development of the bovine embryo
to program postnatal growth and alter muscle DNA methylation. FASEB J. 2021
Oct;35(10):e21926. doi: 10.1096/fj.202100991R. PMID: 34533870.

2: Estrada-Cortés E, Negrón-Peréz VM, Tríbulo P, Zenobi MG, Staples CR, Hansen
PJ. Effects of choline on the phenotype of the cultured bovine preimplantation
embryo. J Dairy Sci. 2020 Nov;103(11):10784-10796. doi: 10.3168/jds.2020-18598.
Epub 2020 Sep 28. PMID: 32896407.manipulating cow nutrition or the composition of
333 culture media used in ART to epigenetically optimize phenotypic outcomes.

**Reviewer 2**

The authors of this paper present the results of numerous statistical analyses of the bovine genome aimed at identifying and analysing specific regions of interindividual epigenetic variability - the so-called CoRSIVs, similar to those identified in the human genome. The study represents a significant advance on previous ones, as it is the first time that analysis of these regions in the bovine genome has been performed. The paper should be of broad interests to others in the field and maybe to a broad audience of biologists. I think that the conclusions of the study are adequately supported by the data shown and the authors provided sufficient details to allow replication and comparison with related analyses. I believe that the methods used are appropriate to the aims of the study and they are well described.

I would just like to make some comments on the formal presentation of the study: it's only my recommendation, but it seems more appropriate to me that:
1. In the conclusion of the Introduction section, the aims of the study should be formulated, not already the results.
2. The legends to the figures should describe the fact in the figure. For example: Fig. 1B: The unsupervised hierarchical clustering of avarage methylation groups by tissue, 1C: The avarage percent of methylation within each 100 bp bin, or Fig. 4A: The overlaping of DMRs associated with methionine supplementation the CoRSIVs and control regions. Or similar ...
I also believe that methods and results should not be presented in the figure legends, these are presented in the relevant sections.

Other comments:
line 161-162: is the sentence correct? Not "are clustered"?
line 179-180: is the sentence correct? Not "is clustered"?
line 182-186: twenty cows used to study the interindividual variation in DNA methylation are not described in more detail, e.g. where they were housed or where they come from.
line 192-194: At the beginning of the section "Independent validation confirms systemic interindividual variation in DNA methylation" the authors state that they used several tissues from 20 cows for the analysis of interindividual variability of DNA methylation. And at the end of this section it is stated that the overall validation rate was somewhat lower than in human studies, likely because only two animals were studied (?)

I would also like to explain the following abbreviations:
line 100: SNP
line 205: SNV
line 234: SIV regions in mice
line 456: DSS - this abbreviation sholud be explained when it is first mentioned and this applies to all abbreviations mentioned in the text

**Reviewer 3**

-The manuscript, by using public available WGBS data, reported the identification of correlated regions of systemic interindividual epigenetic variation (CoRSIVs) in bovine. These findings were only previously described by the same group in human and the methodology utilized is consistent with previous works.
-This is an important topic in veterinary and life science, because it helps to better define animal diversity.
-Animal CpG diversity is reported to be associated with animal genotype and to be enriched in specific repeat elements and it is potentially associated with specific animal traits.
-CoRSIVs blocks identified in silico are also validated by bisulfite pyrosequencing of specific regions.

Major Revision

However, main concern is related to the fact that CpG methylation call after bisulfite treatment (WGBS dataset) is highly influenced by the presence of C/T variation in the genome, and the analysis of animal genetic diversity is required to avoid misinterpretation
Therefore, it is necessary to correctly compute differences in methylation levels by disentangling methylation from genomic variation.
In fact, bisulphite treatment causes the conversion of unmethylated cytosines into uracil, and into thymine during the following PCR reactions. Thymines detected in the bisulphite sequencing experiments are therefore interpreted as not methylated cytosines. For this reason, a distinction between thymines resulting from the deamination of unmethylated cytosines and real thymines, deriving from mutation, was necessary, to clarlfy results from this possible bias. To avoid this problem previous experiment reported the use of 1000 bulls SNPs (Costes et al., 2021), or genotype from same animals (Capra et al., 2022) to correct this bias.
In the WGBS dataset, variation in methylation between the two animals can derive from C→T variations overlapping cytosines, or also in the adjacent cytosine because this would alter the CpG context, recognized by DNA methyltransferase.
For this reason, it is necessary to verifiy that all differentially methylated cytosines (and near ±1bp positions) are real cytosines. This is particularly important when considering positions which methylation does not change in different tissues and could be only dependent on misinterpretation due to SNPs variation. In addition, this could partially explain why you observe a very strong association with genetic variation.

Although, we are confident that CoRSIV methylation can depict animal epigenetic diversity also in cattle, it is not possible to exclude that part of your observation are only due to animal genetic variation.

Did the Authors filter out all cytosine presenting variation in the two animals, (near ±1bp position C/T or G/A SNPs)? If yes, you should clearly explain it in the material and method section (I did not find any explanation in M&M section).

If no, you have to eliminate them. The study from Zhou et al, already provide the WGS dataset from lung of the same animals. https://www.ncbi.nlm.nih.gov/geo/query/acc.cgi?acc=GSE146345

Also the independent validation that you use to confirm the CORVIS could have the same bias due to the presence of polymorphism in those positions.
I will suggest to confirm the absence of SNP polymorphism also in your validation experiment.

Other Minor considerations are:
- Line 112-115: Please provide references
- Line 155: Please provide for each tissue cytosine average coverage per animals
- Line 161: Please specify the percentage of the bovine genome covered
- Line 166: How many blocks?
- Line 186: From supplementary Table 3. The validation seems to be only related to few cytosines in 100bp windows and not for entire blocks. For many of validate bin "block" in S3 is impossible to find the correspondence with S2 bins. Please provide in S3 a column with bin name to understand which cytosines were validate.
- Figure 3C. Color of the bar and legend are inappropriate

**Reviewer 4**

Using publicly data, authors aimed to identify what they called "systemic interindividual epigenetic variation" (CoRSIVs) in cattle. Based on their results they conclude that, as in human, CoRSIVs exist in cattle sugestting these epigenetic variants may be common in mammals. Furthermore, that these variants may be associate to agriculturally important phenotypes and it can be useful for animal breeding .

My comments will be organized by section:

Title:

In my opinion, there is still some confusion in the scientific literature regarding the definition of epigenetics. There is a confusion between epigenetics (it has to be in a context of cell differentiation and transcriptional regulation of gene expression) and epigenetic marks (DNA methylation and post-translational histone modifications). Instead of using "systemic interindividual epigenetic variants" in the title of the manuscript, the most correct would be "systemic interindividual DNA methylation variants", as the manuscript address the DNA methylation mark and not epigenetics.


Introduction:

- Overall, I think the Introduction section was convincing enough to show the relevance of the subject;

- Based on the first paragraph and other statements throughout the text (such as lines 105-106 and 108-110), the authors focus on the potential future application of these epigenetic variants (CoRSIVs) in animal breeding. The authors say that these systemic epigenetic variants are sensitive to the periconceptional environment (lines 124-125; 129-130). In my opinion, the authors do not address at any point in the Introduction section the most obvious and important characteristic of any (epi)genetic marker/variant to be used in the plant or animal breeding, which is whether this marker is inheritable. As the authors themselves state that these epigenetic variants are sensitive to environmental variations (it is possible that the changes induced by the environment do not occur in the germ cells) and that it is also fully established in the literature that epigenetic profiles are erased after fertilization in mammals, how can these epigenetic variants be used for animal breeding whether they may not be inherited? It is essential to address this subject (transgenerational inheritance) here in the Introduction section;

- Pages 139-146: part of this text is Material and Methods and part is Results; please move text to Material and Methods and Results sections;

- Pages 146-147: please move this sentence to Discussion and/or Conclusions sections.


Materials and Methods:

- Authors describe three public dataset they used (references 16, 20 and 21 - lines 352-365). However, throughout the Materials and Methods text they describe the methods used only with data from Zhou et. (for example line 380 - "6 libraries"; line 431 - "...2 subjects x 3 tissues). I guess this information ("6 libraries and 2 subjects x 3 tissues") refers to data from zhou et. al.;

- Lines 406-408: it is necessary explain how authors obtained/collected the samples;

- Lines 412-413 ("Similar to our study in humans [12], a CoRSIV was considered validated if at least one of the three inter-tissue correlations was at least 0.71 (r2 = 0.5)"): please, make it clearer what are these "three inter-tissue correlations". Why do you have three correlations? It's very confusing;

- To test possible environmental effects on methylation profiles in CoRSIVs, why did you choose only methionine supplementation?

- What data were used to test the effects of Assisted Reproductive Technology on CoRSIVs (line 473)? Only in the Results section that this information is presented (lines 257-258);

- To validate the in silico results, authors used liver, kidney and cerebral cortex from calves. However, data they analysed in silico were of front cortex, lung and white blood cells from cows. Why didn't the authors use the same tissues to validate the results?

- Authors themselves consider the data they used are limited, as they state in lines 193-194; 280-281; 303-308; 338-339.


Results:

- Of the 1,387 CoRSIVs that the authors identified, they used 10 to validate the data (Fig. 2 and Fig. S3). Is it correct?

- Please move lines 183-186 to Materials and Methods;

- Please move lines 191-194 to Discussion;

- Please move lines 225-239 to Materials and Methods;

- What does "Supplementation DMRs" (line 242) means?

- I suggest remove the text from lines 251-255. This information is not a result and is not necessary;

- The information "...produced by IVP (n=4) and by MOET..." (line 258) must be presented in Materials and Methods;

- Please move lines 268-270 to Discussion section.


Discussion:

- "...genetic variation..." (line 278): What do the authors mean by genetic variation? SNPs, Indels, etc???

- Lines 297-298: How can a molecular profile that can be susceptible to environmental influences be used in animal selection if it may not be transgenerationally inheritable (for three or more generations)?

- Lines 310-313: CoRSIVs needs to be inheritable (at least for three generations) to be used for animal selection;

- "...is stable for years..." (line 313): What "is stable for years" means? In the same animal or for generations? If the DNA methylation profile of a CoRSIV does not remain stable for different generations it is not useful for animal breeding;

- Line 322: In livestock, infertile or subfertile animals are not included in ART programs;

- Lines 325-326: I didn't understand this sentence. Please, make it clearer;

- Lines 331-333: Changes in DNA methylation profile by manipulating nutrition or culture media can "optimize phenotypic outcomes" in the animal itself or for generations? Please, make it clearer.


Conclusion:

- It is necessary to address here whether the DNA methylation profile in CoRSIVs has inter- or transgenerational inheritance, as this is essential for the use of CoRSIVs in animal breeding.

**Authors’ response to reviewers**

REVIEWER 1
We appreciate the reviewer’s expert feedback and are delighted that s/he found it important to characterize CoRSIVs in cattle. We agree with the reviewer that the major limitation of this study is sample size (n=2; three tissues), which limits the number of CoRSIVs we found (217). However, this number is comparable to our first unbiased WGBS screen in humans that involved two individuals as well, in which we found 109 SIV regions. We have done our best to address all of the reviewer’s suggestions:

Comment: What is missing in this paper is the description of variable sites that are not related to genetic (SNVs) but potentially dependent on the environment and variable in all animals. This is where, having a population of > 12, would be useful as many of the methylation changes observed in defined environments (food/temperature/IVF) are not related to sequences differences and are likely the ones that could provide additional information compared to genotyping.

Response: The reviewer raises an important point that often causes confusion, so we were pleased to add the following lines to the Discussion [LINES 278-283]: “It is important to note that unlike mouse metastable epialleles such as agouti viable yellow and axin-fused, which are generally studied within inbred populations, at CoRSIVs in outbred mammalian populations establishment of DNA methylation is influenced both by periconceptional environment and genetic variation [11-15]. There does not appear to be a dichotomization of CoRSIVs that are influenced by early environment and those associated with genetic variation.”

Comment: The methionine experiment is not well discussed in terms of what additional benefit it provides and why CoRSIVs have the same hypomethylation in methionine supplemented animals.

Response: Because other reviewers also raised questions regarding the methionine experiment, we have excluded it in the revised manuscript. We focused on the ART experiment when investigating the influence of periconceptional environment.

Comment: [Abstract] I would wait for a few more species before making this conclusion: suggesting these systemic epigenetic variants may be common to all mammals

Response: We are not drawing any conclusion, but merely suggesting that CoRSIVs may generally be a common feature of mammalian genomes, which we feel is likely given that these are essentially sites of systemic methylation quantitative trait loci (mQTL). In response to the reviewer’s suggestion we have softened the language further, in both the Abstract [LINES 62-63]: “Our data indicate that CoRSIVs exist in cattle, as in humans, suggesting these systemic epigenetic variants may be common to mammals in general” and in the Conclusion [LINES 339-341]: “More broadly, by suggesting that CoRSIVs may be a feature of mammalian genomes in general, our results indicate that such opportunities for agricultural improvement are not limited to cattle.”

Comment: [Line 119] Do you have evidence in bovine that the blood DNA methylation is a good proxie for the rest of the tissues?

Response: As we mention in Results [LINES 193-197]: “Of the two that showed high inter-tissue correlations in all three comparisons amongst liver, kidney, and brain (Fig. 2), methylation in each of these tissues was also correlated with that in peripheral white blood cells (Fig. S4), indicating that methylation measurements in blood provide a proxy for epigenetic regulation throughout the body, as at human CoRSIVs [15].”

Comment: [Line 265] For the IVP animals, since the methylation changes may be caused by early culture conditions, the CoRSIVs then reflect changes common to all IVP vs MOET and do not represent a genetic programming but potentially a genetic susceptibility to the culture conditions.

Response: We are confused by this comment. The goal of the IVP vs. MOET analyses was to determine if CoRSIVs exhibit heightened sensitivity to early embryonic environment, as observed at human CoRSIVs. We are not trying to demonstrate genetic programming, but rather epigenetic susceptibility to the culture conditions.

Comment: [Line 206] validation study (pyrosequencing or targeted PCR post. conversion) should be done to assess if methylation changes observed at these locus are present in more animals (with different SNVs) for the following genes F2RL3, SIN3B, MACROD2, API5, and PKHD1.

Response: As suggested by Reviewer 3, we re-did the entire CoRSIV screen after filtering out CpG-SNPs, described in the manuscript in Results [LINES 169-175] and Methods [LINES 371-378]. None of the genes named overlap the set of 217 new CoRSIVs in gene bodies. We therefore removed the sentence referring to those genes.

Comment: [Line 325] Please explain the following sentence or justify its value: Hence, CoRSIVs provide a helpful tool when using early embryonic development to guide epigenetic outcome.

Response: We have removed this sentence, and instead elaborated on how methylation profile at CoRSIVs can be used in making culling decisions in the last paragraph in the Discussion [LINE 314-324]: “Importantly, we are not proposing that CoRSIV methylation is transgenerationally heritable, but that it may be a useful variable for optimizing traits within each generation. For example, a potential application for CoRSIVs is to address low female fertility in dairy cattle, by characterizing differences in CoRSIV methylation between individuals with optimal vs. poor fertility. This knowledge could subsequently be used to inform selective breeding practices. During the weaning stage, for example, calves could undergo methylation profiling at CoRSIVs, enabling the identification of female calves with profiles indicative of superior fertility. Selecting such individuals for further breeding could potentially improve overall reproductive efficiency within dairy cattle populations, promoting sustainable dairy farming. For example, in a rat model, GIMAP7 (Fig. 2B) was recently shown to promote oxidative stress and apoptosis in ovarian granulosa cells [27].”

Comment: [Line 333] This supposition has already been demonstrated and please provide references.

Response: We appreciate the reviewer suggesting these salient references. We have cited them as references #28 and #29 at the end of Discussion [LINE 327].

REVIEWER 2
We appreciate the reviewer’s expert feedback and are delighted that s/he recognized that this paper “should be of broad interests to others in the field” and “represents a significant advance on previous ones”. We have done our best to address all of the suggestions:

Comment: In the conclusion of the Introduction section, the aims of the study should be formulated, not already the results.

Response: We have removed description of results in the Introduction. The Introduction now concludes [LINES 140-144]: “Here, by reanalyzing the data of Zhou et al [16], we screened for cattle CoRSIVs. At a subset of regions, systemic interindividual variation was validated by bisulfite pyrosequencing in an independent sample of Holstein cattle (males and females). We also evaluated major hallmarks of human CoRSIVs: association with genetic variation and sensitivity to periconceptional environment.”

Comment: The legends to the figures should describe the fact in the figure. I also believe that methods and results should not be presented in the figure legends, these are presented in the relevant sections.

Response: We have eliminated all details on methods and p values from main figure legends. We have also revised all figure legends to succinctly describe what is shown in the figure. For example, in Fig. 1B: “Unsupervised hierarchical clustering of average methylation groups by tissue (a random sample of 30,000 genomic bins is shown)”; and in Fig. 1C: “Illustration of our two-step approach: an example of a 300 bp block with concordant positive residuals is indicated by the red box”; and in Fig. 4B: “Overlap of differentially methylated regions (DMRs) between IVP and MOET offspring with CoRSIVs and control in three tissues: liver, muscle, and hypothalamus.”

Comment: [Line 161-162] is the sentence correct? Not "are clustered"?

Response: To improve clarity, we have reworded the sentence to “unsupervised hierarchical clustering of average CpG methylation in a randomly selected subset of 30,000 grouped by tissue” [LINE 157-159].

Comment: [Line 179-180] is the sentence correct? Not "is clustered"?

Response: To improve clarity, we have reworded the sentence to “unsupervised hierarchical clustering of average CpG methylation at these 217 CoRSIVs grouped by individual” [LINE 185-186].

Comment: [Line 182-186] twenty cows used to study the interindividual variation in DNA methylation are not described in more detail, e.g. where they were housed or where they come from.

Response: We have now included a description on the twenty cows in Methods [LINE 414-418]: “The calves were housed with standard care at the Cornell University Ruminant Center (Harford, NY) and transported to the abattoir of Frank Morrison Hall (Ithaca, NY) on the day of tissue collection. Euthanasia was performed by penetrating captive bolt followed by exsanguination during which blood was collected. Immediately following euthanasia, different types of tissues were collected from each calf and snap frozen on dry ice.”

Comment: [Line 192-194] At the beginning of the section "Independent validation confirms systemic interindividual variation in DNA methylation" the authors state that they used several tissues from 20 cows for the analysis of interindividual variability of DNA methylation. And at the end of this section it is stated that the overall validation rate was somewhat lower than in human studies, likely because only two animals were studied (?)

Response: We thank the reviewer for pointing out this potential point of confusion. We used 20 animals in the validation process, where we performed pyrosequencing on a subset of the 217 CoRSIVs. The initial CoRSIV screen was based on WGBS data of only two cows. We have clarified the exact number of cows used in the screen and validation experiment in LINE 148-150 (for the screen), and LINE 189-190 (for validation).

Comment: Abbreviations for SNP, SNV, SIV, DSS.

Response: We have added full terminology at first occurrence of all abbreviations: single-nucleotide polymorphism (SNP) [LINE 99], systemic interindividual variation (SIV) [LINE 119], Dispersion Shrinkage for Sequencing data (DSS) [LINE 481]. SNV is no longer used in the manuscript.

REVIEWER 3
We appreciate the reviewer’s expert feedback and are delighted that s/he recognized that “this is an important topic in veterinary and life science” and “helps to better define animal diversity”. In particular, we appreciate his/her insightful suggestion regarding disentangling methylation from genetic variation by filtering out CpGs affected by SNPs. In response to this suggestions, we re-performed the entire CoRSIV screen after masking such affected CpGs, and conducted additional validation experiments based on the filtered coverage file free of genetic variations. We have done our best to address all of the reviewer’s suggestions:

Comment: filter out all cytosine presenting variation in the two animals, (near ±1bp position C/T or G/A SNPs) using WGBS data from lung of the same animals

Response: We performed this filtering, as now described in Methods [LINES 374-378]: “Using genotyping data published by Zhou et al. on the lung tissue of the two Holstein cows used initially in the screen, we identified 842,724 discordant SNPs affecting either a C or G in a CpG site with quality score ≥ 30, which were subsequently removed from the coverage file and all subsequent methylation analysis.” In Results [LINE 168-175], we compared the two sets of CoRSIVs before and after filtering out CpG-SNPs. Filtering out SNPs drastically reduced the number of CoRSIVs we identified (from 1387 to 217). As we now mention in Discussion [LINES 289-291], this number is actually more consistent with our first human study, which was based on two individuals. While a subset of CpGs within the previous set of CoRSIVs may be impacted by discordant SNPs, leading to artefactual methylation differences, other CpGs within these regions may indeed reflect genuine interindividual differences, so we view this masking approach as highly conservative. It is plausible that a substantial proportion of the ‘false-positive’ CoRSIVs we previously identified could be recovered with increased sample size and deeper coverage.

Importantly, although the number of regions identified is much lower than in the original manuscript, we were able to show that the current set of CoRSIVs is still associated with genetic variation and sensitive to periconceptional environment, specifically in the IVP vs. MOET analyses.

Comment: Confirm the absence of SNP polymorphism also in validation experiment using 1000 Bull SNPs

Response: We also appreciate this astute suggestion. As now described in Methods [LINES 355-359]: “we combined run8 and run9 of the 1000 Bull Genomes Project and included 254 Holstein samples. We only included variants with valid GT in at least 200 Holstein samples and minor allele frequency ≥ 0.05 in all subsequent analysis.” Also as now described in Methods [LINES 419-422]: “when designing pyrosequencing assays, data on common SNPs in Holstein cattle in the 1000 Bull Genomes Project [20] were used to ensure that SNPs neither overlapped the PCR or sequencing primers, nor were within the sequence analyzed.”

Comment: [Line 112-115] Please provide references

Response: Reference #8 inserted in Introduction [LINE113].

Comment: [Line 155] Please provide for each tissue cytosine average coverage per animals

Response: Coverage per sample, as well as other sample information, are included in Table S1.

Comment: [Line 161] Please specify the percentage of the bovine genome covered

Response: We have included this information in the Methods [LINE 382-384]: “We focus on the ~ 10.5M 100 bp bins with adequate coverage (roughly 83% of all ~12.6M 100bp bins containing at least one CpG) in at least two tissues in each animal.”

Comment: [Line 166] How many blocks?

Response: We have included this information in the Methods [LINE 390-391]: “We identified 138,192 genomic blocks comprised of two or more consecutive bins with residuals having absolute values ≥ 10%, in the same direction.”

Comment: [Line 186] From supplementary Table 3. The validation seems to be only related to few cytosines in 100bp windows and not for entire blocks. For many of validate bin "block" in S3 is impossible to find the correspondence with S2 bins. Please provide in S3 a column with bin name to understand which cytosines were validate.

Response: The table on pyrosequencing is now Table S5. Table S5 is updated to include Assay Bin Name (Based on Column A in S3 CoRSIV list), CoRSIV ID (Based on Column AE in S3 CoRSIV list), CoRSIV coordinates, and validation status for each region.

Comment: Figure 3C, Color of the bar and legend are inappropriate

Response: We corrected the figure legend's color to reflect proper CoRSIV vs control group membership.

REVIEWER 4
We appreciate the reviewer’s expert feedback and suggestions for improving our manuscript. In particular, we appreciate the reviewer’s comment on transgenerational inheritance as a potential source of confusion in our paper. We have done our best to address all of the suggestions:

Comment: Instead of using "systemic interindividual epigenetic variants" in the title of the manuscript, the most correct would be "systemic interindividual DNA methylation variants", as the manuscript address the DNA methylation mark and not epigenetics.

Response: We have changed the title as suggested.

Comment: In my opinion, the authors do not address at any point in the Introduction section the most obvious and important characteristic of any (epi)genetic marker/variant to be used in the plant or animal breeding, which is whether this marker is inheritable. As the authors themselves state that these epigenetic variants are sensitive to environmental variations (it is possible that the changes induced by the environment do not occur in the germ cells) and that it is also fully established in the literature that epigenetic profiles are erased after fertilization in mammals, how can these epigenetic variants be used for animal breeding whether they may not be inherited?

Response: We appreciate this comment, which points to an issue that other readers might likewise find confusing. In response, we included in the Discussion a description on how CoRSIVs could potentially be used in making culling decisions [LINE 311-324]: “In addition to genetic profiling, we have identified an additional level of individuality at the epigenetic level. Consideration of epigenetic variation at CoRSIVs may provide a useful complement to management strategies based on genotyping [3]. Importantly, we are not proposing that CoRSIV methylation is transgenerationally heritable, but that it may be a useful variable for optimizing traits within each generation. For example, a potential application for CoRSIVs is to address low female fertility in dairy cattle, by characterizing differences in CoRSIV methylation between individuals with optimal vs. poor fertility. This knowledge could subsequently be used to inform selective breeding practices. During the weaning stage, for example, calves could undergo methylation profiling at CoRSIVs, enabling the identification of female calves with profiles indicative of superior fertility. Selecting such individuals for further breeding could potentially improve overall reproductive efficiency within dairy cattle populations, promoting sustainable dairy farming. For example, in a rat model, GIMAP7 (Fig. 2B) was recently shown to promote oxidative stress and apoptosis in ovarian granulosa cells [27].”

Comment: [Lines 139-146] part of this text is Material and Methods and part is Results; please move text to Material and Methods and Results sections

Response: We have moved the more detailed descriptions of the methods and datasets to the appropriate Materials and Methods section. The Introduction now concludes [LINES 140-144]: “Here, by reanalyzing the data of Zhou et al [16], we screened for cattle CoRSIVs. At a subset of regions, systemic interindividual variation was validated by bisulfite pyrosequencing in an independent sample of Holstein cattle (males and females). We also evaluated major hallmarks of human CoRSIVs: association with genetic variation and sensitivity to periconceptional environment.”

Comment: Authors describe three public dataset they used (references 16, 20 and 21 - lines 352-365). However, throughout the Materials and Methods text they describe the methods used only with data from Zhou et. (for example line 380 - "6 libraries"; line 431 - "...2 subjects x 3 tissues). I guess this information ("6 libraries and 2 subjects x 3 tissues") refers to data from zhou et. al.;

Response: We have clarified the dataset we used for the ART analyses in Results [LINES 234-236]: “Rabaglino et al. [19] obtained samples from multiple tissues from three-month-old calves produced by IVP (n=4) and by MOET (control, n=4) and performed WGBS. We analyzed their WGBS data” and Methods [LINE 479]: “Using WGBS data generated by Rabaglino et al. [19], we called Bismark methylation extractor on BAM files and converted coverage files...”.

Comment: [Lines 146-147] please move this sentence to Discussion and/or Conclusions sections.

Response: We have removed this sentence from Introduction.

Comment: [Lines 406-408] it is necessary explain how authors obtained/collected the samples;

Response: We have now included these details in Methods [LINE 416-418]: “Euthanasia was performed by penetrating captive bolt followed by exsanguination during which blood was collected. Immediately following euthanasia, different types of tissues were collected from each calf and snap frozen on dry ice.”

Comment: [Lines 412-413] ("Similar to our study in humans [12], a CoRSIV was considered validated if at least one of the three inter-tissue correlations was at least 0.71 (r2 = 0.5)"): please, make it clearer what are these "three inter-tissue correlations". Why do you have three correlations? It's very confusing;

Response: We appreciate this suggestion, which made us realize we had not introduced the terminology “systemic interindividual variation”, which we now do in the Introduction [LINEs 118-119] “Such epigenetic variation is called systemic interindividual variation (SIV).” Also, we now state in Methods [LINES 426-429] “High inter-tissue correlation (for example, liver vs. kidney) is a hallmark of SIV. Similar to our study in humans [12], a CoRSIV was considered validated if at least one of the three inter-tissue correlations (liver-kidney, kidney-cortex, liver-cortex) was at least 0.71 (r2 = 0.5).”

Comment: To test possible environmental effects on methylation profiles in CoRSIVs, why did you choose only methionine supplementation?

Response: We have now excluded the methionine experiment in our revised manuscript, as multiple reviewers raised questions about it.

Comment: What data were used to test the effects of Assisted Reproductive Technology on CoRSIVs (line 473)? Only in the Results section that this information is presented (lines 257-258);

Response: We have clarified the dataset we used for ART experiment in Methods [LINE 479]: “Using WGBS data generated by Rabaglino et al. [19], we called Bismark methylation extractor on BAM files and converted coverage files...”.

Comment: To validate the in silico results, authors used liver, kidney and cerebral cortex from calves. However, data they analysed in silico were of front cortex, lung and white blood cells from cows. Why didn't the authors use the same tissues to validate the results?

Response: It is generally advantageous if the tissues used for validation are somewhat different from those used for the initial screen. To confirm the systemic nature of CoRSIVs, we intentionally picked different tissues from those used in the initial screen, one for each germ layer. Also, nearly all of our previous studies have used liver, kidney, and brain to represent the three germ layers in validation studies. Justification is now included in Methods [LINE 412-414]: “The sets of tissues representing the germ layers were chosen to be different in the validation experiment rather than in the initial screen, to better test the systemic nature of interindividual variation at CoRSIVs.”

Comment: Of the 1,387 CoRSIVs that the authors identified, they used 10 to validate the data (Fig. 2 and Fig. S3). Is it correct?

Response: Per Reviewer #3’s suggestions, we have reperformed the entire screen after filtering out genetic variations and performed extra validation experiments, so the number of CoRSIVs and the number of regions chosen for validation have now changed. The details are now updated in Results [LINES 189-190]: “We obtained liver, kidney, and cerebral cortex of 20 Holstein calves and selected 11 of the 217 CoRSIVs for validation by pyrosequencing, as in our previous studies [11, 12] (Fig. 2; Table S5).”

Comment: Please move lines 183-186 to Materials and Methods;

Response: We have moved this text to Materials and Methods and kept a short general summary about validation experiment in its place, Results [LINES 189-190]: “We obtained liver, kidney, and cerebral cortex of 20 Holstein calves and selected 11 of the 217 CoRSIVs for validation by pyrosequencing, as in our previous studies [11, 12] (Fig. 2; Table S5).”

Comment: Please move lines 191-194 to Discussion;

Response: The correlation between blood with three other tissues are a part of our Results, so we chose to keep that unchanged. We have moved the text regarding validation rate to Discussion [LINE 292-294]: “Likely due to this limited sample size and sequencing depth, the overall validation rate was somewhat lower than in our human studies [14].”

Comment: Please move lines 225-239 to Materials and Methods;

Response: We have reworded them and moved them to Methods [LINES 487-490]: “Because of the relatively low number of DMR overlaps identified, as a complementary approach, we performed a read-level analysis to identify CoRSIVs or control regions showing statistically significant differences in methylation associated with IVP vs. MOET group.”

Comment: What does "Supplementation DMRs" (line 242) means?

Response: Supplementation DMRs are DMRs between supplemented group and control group. However, this is no longer discussed in the revised manuscript, as the methionine experiment is now excluded from the manuscript.

Comment: I suggest remove the text from lines 251-255. This information is not a result and is not necessary;

Response: We respectfully disagree. This information helps establish the context for the following dataset, and we believe it is helpful to include a brief introduction to ART here.

Comment: The information "...produced by IVP (n=4) and by MOET..." (line 258) must be presented in Materials and Methods;

Response: We have added these details in Methods [LINES 350-352]: “Rabaglino et al. [19] obtained samples from 6 tissues from three-month-old calves produced by IVP (n=4) and by MOET (control, n=4) and performed WGBS (GEO accession number: GSE223098)”.

Comment: Please move lines 268-270 to Discussion section.

Response: We have chosen to keep these in the Results section, since methylation level at DMRs overlapping CoRSIVs are a part of the results we are reporting.

Comment: ...genetic variation... (line 278): What do the authors mean by genetic variation? SNPs, Indels, etc???

Response: Both SNPs and Indels, which we now clarify by adding in Results [LINES 211-213]: “We therefore analyzed genotyping data of Zhou et al. [16], including both SNPs and insertions/deletions (indels), on the two cows in our screen” and Methods [LINE 466]: “…overlapping discordant and concordant genetic variations (including both SNPs and indels)…”.

Comment: [Lines 297-298] How can a molecular profile that can be susceptible to environmental influences be used in animal selection if it may not be transgenerationally inheritable (for three or more generations)?

Response: We thank the reviewer for pointing out this potential source of confusion. By selection, we mean when making culling decisions, not genetic selection. We now include a clarification in Discussion [LINES 319-323]: “During the weaning stage, for example, calves could undergo methylation profiling at CoRSIVs, enabling the identification of female calves with profiles indicative of superior fertility. Selecting such individuals for further breeding could potentially improve overall reproductive efficiency within dairy cattle populations, promoting sustainable dairy farming.”

Comment: [Lines 310-313] CoRSIVs needs to be inheritable (at least for three generations) to be used for animal selection;

Response: By selection, we mean when making culling decisions, not genetic selection. We addressed this in the previous comment.

Comment: "...is stable for years..." (line 313): What "is stable for years" means? In the same animal or for generations? If the DNA methylation profile of a CoRSIV does not remain stable for different generations it is not useful for animal breeding;

Response: We have clarified this in the Discussion to avoid confusion about genetic selection in breeding programs [LINES 301-304]: “Human studies have shown that methylation at CoRSIVs is stable for years in the same individual [12, 22, 23]. It is possible, therefore, that profiling CoRSIV methylation in peripheral blood DNA of calves could someday be utilized to identify those individuals most likely to exhibit traits favorably associated with productivity in adulthood.”

Comment: [Line 322] In livestock, infertile or subfertile animals are not included in ART programs;

Response: We have excluded this sentence from our revised manuscript.

Comment: [Lines 325-326] I didn't understand this sentence. Please, make it clearer;

Response: We have removed this sentence.

Comment: [Lines 331-333] Changes in DNA methylation profile by manipulating nutrition or culture media can "optimize phenotypic outcomes" in the animal itself or for generations? Please, make it clearer.

Response: We have clarified in the Discussion that we are referring to within one generation, and have included two references as suggested by another reviewer [LINES 324-327]: “Beyond such selection for desirable traits within each generation, our results on periconceptional influence, specifically embryo culture during IVP, suggest the possibility of manipulating cow nutrition or the composition of culture media used in IVP to epigenetically optimize phenotypic outcomes [28, 29].”

Comment: It is necessary to address here whether the DNA methylation profile in CoRSIVs has inter- or transgenerational inheritance, as this is essential for the use of CoRSIVs in animal breeding.

Response: We appreciate the reviewer’s suggestion to clarify the issue of transgenerational inheritance, which we now do in the Discussion [LINES 314-316]: “Importantly, we are not proposing that CoRSIV methylation is transgenerationally heritable, but that it may be a useful variable for optimizing traits within each generation.”

**Second round of review**

**Reviewer 1**

Thanks for replying to my questions and the modifications you made to the manuscript

**Reviewer 3**

Authors responded to all my revisions

**Reviewer 4**

The authors responded satisfactorily to all my comments and questions. Therefore, in my opinion, the revised manuscript can be accepted for publication in Genome Biology
